# Genetic variation architecture of mitochondrial genome reveals the differentiation in Korean landrace and weedy rice

**DOI:** 10.1038/srep43327

**Published:** 2017-03-03

**Authors:** Wei Tong, Qiang He, Yong-Jin Park

**Affiliations:** 1 Department of Plant Resources, College of Industrial Science, Kongju National University, Yesan, 32439, Republic of Korea; 2 State Key Laboratory of Tea Plant Biology and Utilization, Anhui Agricultural University, Hefei, 230036, Peoples’ Republic of China; 3 Center for crop genetic resource and breeding (CCGRB), Kongju National University, Cheonan, 31080, Republic of Korea

## Abstract

Mitochondrial genome variations have been detected despite the overall conservation of this gene content, which has been valuable for plant population genetics and evolutionary studies. Here, we describe mitochondrial variation architecture and our performance of a phylogenetic dissection of Korean landrace and weedy rice. A total of 4,717 variations across the mitochondrial genome were identified adjunct with 10 wild rice. Genetic diversity assessment revealed that wild rice has higher nucleotide diversity than landrace and/or weedy, and landrace rice has higher diversity than weedy rice. Genetic distance was suggestive of a high level of breeding between landrace and weedy rice, and the landrace showing a closer association with wild rice than weedy rice. Population structure and principal component analyses showed no obvious difference in the genetic backgrounds of landrace and weedy rice in mitochondrial genome level. Phylogenetic, population split, and haplotype network evaluations were suggestive of independent origins of the *indica* and *japonica* varieties. The origin of weedy rice is supposed to be more likely from cultivated rice rather than from wild rice in mitochondrial genome level.

The mitochondrial (mt) genome plays an essential role in cell metabolism. Plant mt genomes vary greatly in size, from ~200 kb to 2 Mb mostly, and are substantially larger than mt genomes of other eukaryotes (https://www.ncbi.nlm.nih.gov/genome/browse/?report=5#)^1^,^2^. Some specialized plant (like *Silene conica*) even has a genome size over 10 Mb^3^. Physical mapping and sequencing of some of the small mt genomes show that their structures are shaped by active recombination, gene transfer to the nucleus, and other forces that remain unclear^4^. Structural analyses revealed high frequencies of intra- and intermolecular recombination, which generated a structurally dynamic assemblage of genome configurations^5^,^6^. This dynamic organization of the plant mt genome provides a powerful model for the study of genome structure and evolution. These genomes exhibit an intriguing mixture of conservative (slowest rates of nucleotide substitution)^7^,^8^ and dynamic evolutionary patterns. Some previous studies^9^,^10^,^11^,^12^ also suggested that for evolutionary studies it is not necessary to assemble whole organelle genomes but just exploring the variations.

The complete chloroplast and mt genomes of rice are available^2^,^13^,^14^,^15^, and comparative analysis showed that the gene order and essential gene content are highly conserved for most chloroplast genomes. In contrast, mt-encoded genes are highly conserved, but their gene order, genome structure, and genome size are highly variable among plant species^2^,^16^. In rice, the intersubspecific polymorphism rate for mitochondrial genome is 0.02% for SNPs (single nucleotide polymorphisms) and 0.006% for indels (insertions and deletions) and the intravarietal polymorphism rates among mitochondrial genomes are about 1.3% for SNPs and 1.1% for indels, respectively. Some intravarietal polymorphisms are fixed in *indica* or *japonica*, which can be used as specific markers in distinguishing the two subspecies^2^. Whole-organelle genome sequencing, especially for the chloroplast and mt genomes, has been applied recently as a potential barcode^17^ that can assist in overcoming the previous process of collecting data over generations. Furthermore, due to recombination in the nucleus, data may lead to the construction of unreliable phylogenies; organelles are structurally stable, non-recombinant, and haploid, and thus offer certain advantages in phylogenetic reconstruction.

Asian cultivated rice (*Oryza sativa* L.) is generally thought to have been domesticated from *O. rufipogon* several thousands of years ago^18^,^19^,^20^,^21^. However, some debate regarding the origin of cultivated rice has emerged over the past several years, centering on whether the two major rice cultivars, *O. sativa* ssp. *indica* and *japonica*, were derived from a single ancestor or were domesticated independently at different locations^10^,^19^,^22^,^23^,^24^,^25^. As a consequence of adaptation to different habitats, extensive genotypic and phenotypic diversity exists within *O. sativa* L., resulting in about 120,000 different accessions^20^. These accessions range from traditional rice landraces preserved by indigenous farmers to the commercially bred cultivars developed during the green revolution. Landraces are local varieties of a domesticated plant species that were adapted to their natural and cultural environments. Rice landraces are lineages developed by farmers through artificial selection during the long-term domestication process. Each landrace has particular properties or characteristics, such as early maturity, adaptation to particular soil types, resistance or tolerance to biotic and abiotic stresses, and properties related to the expected end usage of the grains. Exploring the genetic basis of these diverse varieties will provide important insight for the breeding of elite varieties for sustainable agriculture.

Weedy rice (*Oryza sativa* f. *spontanea* Rosh.), harbors characteristics of undomesticated *Oryza* species, including seed dispersal mechanisms and seed dormancy. It also possesses traits of domesticated rice, such as rapid growth, and resembles domesticated rice during the seedling stage, which promotes its invasiveness in the agroecosystem^26^,^27^. The origin of weedy rice has long been discussed, which has led to several hypotheses, including the ongoing selection and adaptation of wild rice^28^,^29^, hybridization between cultivated rice and its progenitor type^30^, hybridization between *indica*-*japonica*^26^, ongoing and multidirectional hybridization between weedy rice and cultivated types, and among weedy types^31^. Simple sequence repeats based analyses of the genetic diversity of weedy rice populations from China suggested that weedy rice most likely originated from local *indica* or *japonica* varieties^27^,^32^. In Korea, weedy rice varieties have been collected from farmers’ fields, and their regional distribution and genetics have been characterized extensively^33^,^34^. As weedy rice is a member of the *Oryza* genus, gaining an understanding of the genetic background of problematic weedy species by examining the underlying genomic information is important.

In the present study, a collection of 60 Korean landrace and weedy rice, and 10 wild rice varieties, including *O. sativa* L. ssp. *indica* and *japonica, O. rufipogon*, and *O. nivara*, were selected to investigate the rice mt genome architecture. The mt genome of *O. sativa* L. ssp. *japonica* (Nipponbare, Genbank: NC_011033) was chosen as the reference for mapping variations in the collection. Mt genome variations in the germplasm were mined and subjected to comparative analyses among different groups (wild and the others; *indica, japonica*, and wild; as well as landrace and weedy). The diversity and population structure of these accessions were also examined at the mt genome level. Phylogenetic analyses were performed using the maximum likelihood (ML) and Bayesian inference (BI) methods, together with population split and haplotype network analyses, which could reveal phylogenetic relationships among landrace and weedy rice, and possibly the origin of weedy rice. This report provides a case study for the rice mt genome developed from whole genome resequencing, and the data generated here could be applied to further analyses of rice mt evolution and genetics.

## Results

### Mt variations across the genome

**Figure 1:**
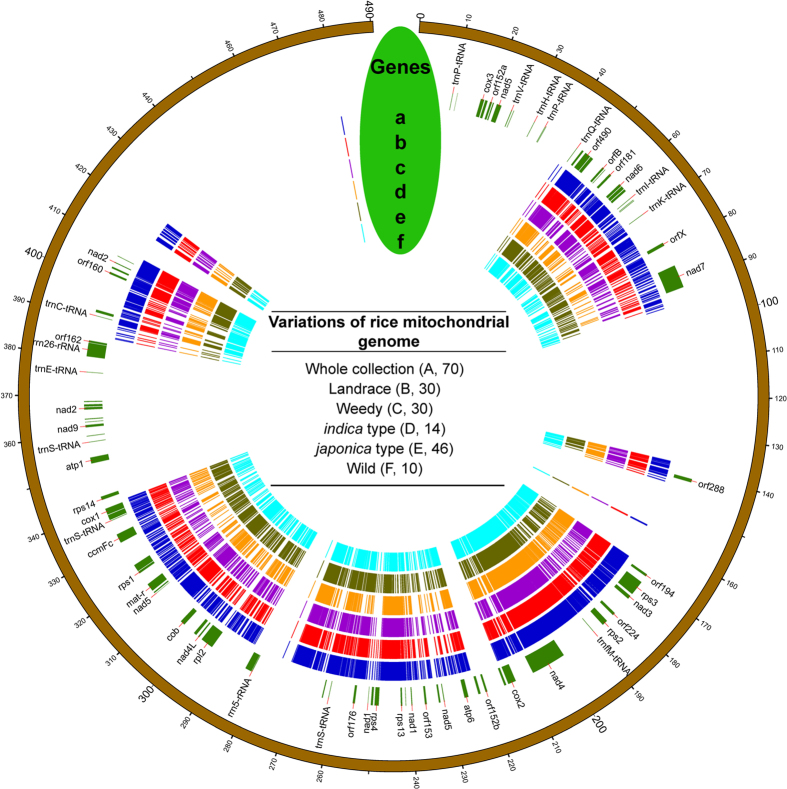
Overall variation (SNPs and Indels) distribution across the mt genome. Genes indicating the genes of the mt genome based on the reference of *O. sativa japonica*. (**a**–**f**) Highlights marked on the circle map revealing the SNP/indel positions. (**a**) Total variations detected in 70 accessions, (**b**) Variations in Korean landrace rice, (**c**) Variations in Korean weedy rice, (**d**) Variations in the rice of *indica* type, (**e**) Variations in *japonica* type, (**f**) Variations in wild rice. The unit of the outside distance is kb. The number inside the brackets indicated the accession numbers of each subgroup. In case of the space, not all the genes were illustrated in the figure.

A number of variations in the rice mt genome were observed in the current study. In total, 4,717 variations, including 4,507 SNPs and 210 indels, were identified across the mt genomes of landrace and weedy rice accessions, along with wild rice. However, after removing missing values and heterozygotes, only 264 high-quality (HQ) variations remained (Table 1). Excluding wild rice, we identified 3,960 variations across the landrace and weedy rice accessions, with 203 HQ sites (Supplementary Tables 1 and 2). In total, 3041 variations (76.8% of total) among the 60 rice accessions located in the intergenic regions, while only 23.2% variations are in the gene region (Supplementary Table 1). When we assigned these variations to different groups, excluding the total SNPs, no significant difference was observed in the distribution of variations between landrace and weedy rice (Table 1). The overall distribution of variations in the total collection and different groups was also targeted based on the reference genome (Fig. 1), which suggested that the variations occurred with a region-dependent preferential. Few variations were observed in several large regions of the genome, some of which contained no variation (Supplementary Table 3).

### Variation architecture at the mt genome level

**Figure 2:**
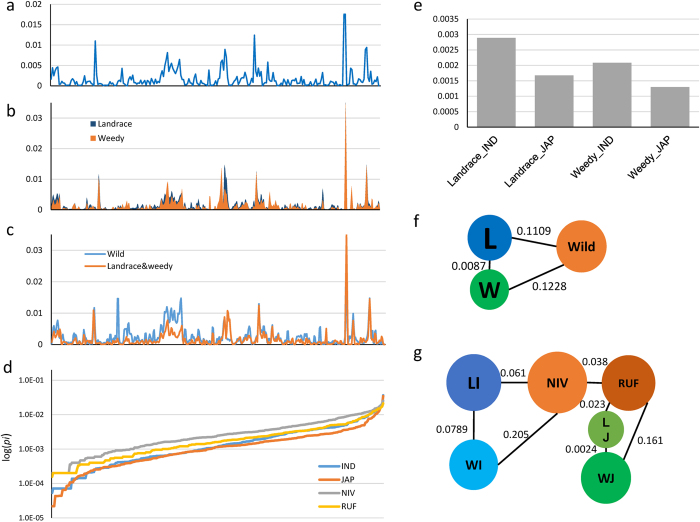
Mitochondrial genome nucleotide diversity (*pi*) and genetic distance (*Fst*). (**a**–**c**) *pi* of the whole collection; landrace and weedy rice; wild and the others (landrace and weedy rice). (**d**) The *pi* of *indica, japonica, O. nivara* and *O. rufipogon*. Values are sorted by ascending. (**e**) The average *pi* of *indica* and *japonica* in landrace or weedy rice. (**f**,**g**) The *Fst* between different groups. The circles indicated different groups and the circle size indicated the *pi* value. The *Fst* value between each two groups were represented by the distance between them. L: landrace rice, W: weedy rice, Wild: wild rice. LI: *landrace*_*indica*, WI: *weedy*_*indica*, LJ: *landrace*_*japonica*, WJ: *weedy*_*japonica*, NIV: *O. nivara*, RUF: *O. rufipogon*.

The nucleotide diversity (*pi*) of the mt genome in the whole collection and different subgroups (landrace, weedy and wild rice, and *indica* and *japonica*) was calculated (Fig. 2). In the whole collection, *pi* ranged from 0.0176 to 8.23E-06 with 1 kb slide window among whole mt genome. Most of the *pi* values were lower than 0.005 (Fig. 2a, Supplementary Table 4), while some regions with extremely high diversity were also identified. In most regions of the mt genome, landrace rice showed slightly greater diversity than weedy rice (Fig. 2b, Supplementary Table 5). Wild rice showed higher diversity than landrace and weedy rice at most mt genome regions(Fig. 2c, Supplementary Table 6). By sorting the *pi* values for *O. rufipogon, O. nivara*, and *O. japonica* and *O. indica*, we found that *O. rufipogon* had the greatest diversity and *japonica* had the least diversity among the four groups (Fig. 2d, Supplementary Table 7). The average *pi* of the *indica* and *japonica* types was greater in landrace rice than in weedy rice (Fig. 2e, Supplementary Table 8). These results were also suggestive of greater diversity in *indica* than in *japonica*. The genetic distance (*Fst*) among the different groups was also calculated, which revealed a very high level of breeding (low *Fst* value) between landrace and weedy rice, and a closer association between landrace rice and wild rice (0.1109) than weedy rice and wild rice (0.1228) (Fig. 2f). When we isolated the wild rice into *O. rufipogon* and *O. nivara*, and the landrace and weedy rice into *indica* and *japonica* (Fig. 2g), the *weedy*_*indica* was more distant with *O. nivara* than *landrace*_*indica*, indicating that *weedy*_*indica* had much lower level of breeding with *O. nivara* than the *landrace*_*indica*. Similar results were observed among the *O. rufipogon* and *japonica* types (landrace and weedy), illustrating that the *landrace*_*japonica* was much closer with *O. rufipogon* than *weedy_japonica*. However, the breeding level between *weedy*_*japonica* and *landrace*_*japonica* was much higher than between *weedy*_*indica* and *landrace*_*indica*. In addition, overall *Fst* values between *O. rufipogon* and *japonica* type were lower than those between *O. nivara* and the *indica* type in landrace and weedy rice.

### Admixed population structure of landrace and weedy rice

**Figure 3:**
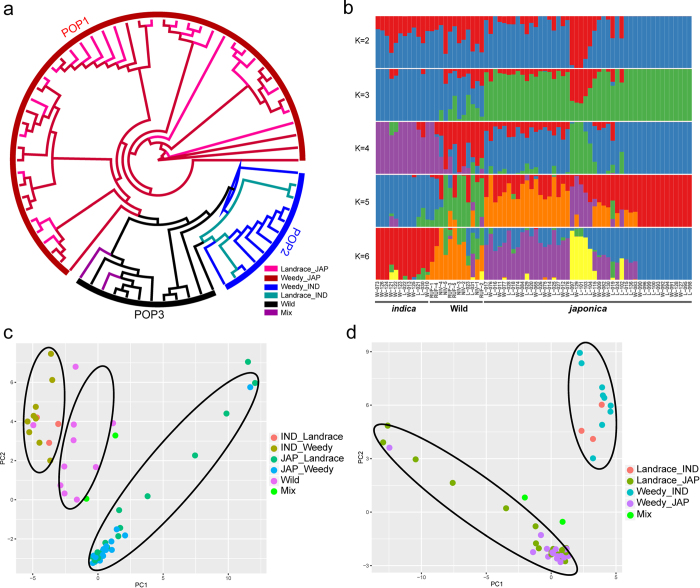
Population structure and principal component analysis of the collection. (**a**) A neighbor-join tree reveals the phylogenetic of the collection. There main populations were identified. The landrace (*indica* and *japonica* type), weedy (*indica* and *japonica* type) and wild rice are marked with different color. (**b**) Population structure clustering of the collection with increasing *K* value from 2 to 6. (**c**,**d**) Principal component analysis of all the accessions (with wild rice) and only landrace and weedy rice accessions. The landrace (*indica* and *japonica* type), weedy (*indica* and *japonica* type) and wild rice are marked with different color. IND: *indica*, JAP: *japonica*, Mix indicated the two accessions in landrace which are mixed with wild rice.

A neighbor-joined phylogenetic tree was constructed in PHYLIP with 1,000 replicates based on the mt genome SNPs, and the consensus tree is shown in Fig. 3a. As illustrated, the *japonica, indica*, and wild types fell into three subpopulations (POP1, POP2, and POP3, respectively). However, the landrace and weedy *indica* types were mixed, as were the landrace and weedy *japonica* types. This admixture was also observed in the population structure estimation with increasing *K* (number of populations) values from 2 to 6 (Fig. 3b). When the *K* value increased to 6, the clear separation of *indica* and *japonica* types was preserved; however, no obvious clustering of *landrace_indica* and *weedy_indica* be detected in *indica* group, similar to *landrace_japonica* and *weedy_japonica* in *japonica* group. PCAs (principal component analysis) of the population with and without wild rice were also performed to compare the grouping of the different groups, which revealed that the *indica, japonica*, and wild types could be grouped (Fig. 3c). However, the landrace or weedy *indica* and *japonica* types were mixed together (Fig. 3d), which was consistent with the results from the neighbor-join phylogenetic and population structure analyses.

### Phylogenetic relationships in landrace and weedy rice

**Figure 4:**
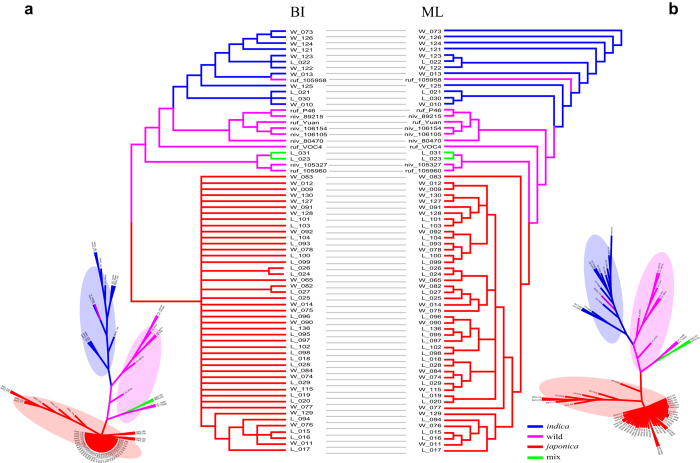
A tanglegram phylogenetic analysis using trees from ML and BI methods to compare the differences from two methods. (**a**) Phylogram and radial tree layout of the ML tree. (**b**) BI-based tree using the same datasets. Best-fit models were evaluated using jModeltest. The tanglegram was implemented in Dendroscope using a Neighbor Net-based heuristic method, which use line connects the same accession in two trees to see the difference phylogenetic structure. *indica, japonica*, wild and mix groups were marked with different colours. The accessions named with “L” or “W” indicate the landrace and weedy rice.

Phylogenetic analysis of the whole collection was performed using a Bayesian MCMC search with MrBayes 3.2.5 software using the best-fit model K80 (Fig. 4a). In parallel, a ML iterative model-based method (with best-fit model SYM) with a bootstrap of 1,000 replicates to assess the reliability of the phylogeny reconstructed using PhyML was also conducted (Fig. 4b). A tanglegram was then constructed for the two trees generated using the two methods, which showed complete consistency with three major clusters: *indica, japonica*, and wild types. Although the tree topology structure generated by the two methods differed, the phylogenetic relationships of these accessions were similar. Wild rice was located in the middle with *indica* and *japonica* in the two opposite sides, suggesting that *indica* and *japonica* may have different origins. In addition, we found no clear separation of landrace and weedy rice within the *indica* and *japonica* types, which is indicative of their sophisticated genetic backgrounds.

### Haplotype network and population splits based on the mt genome

**Figure 5:**
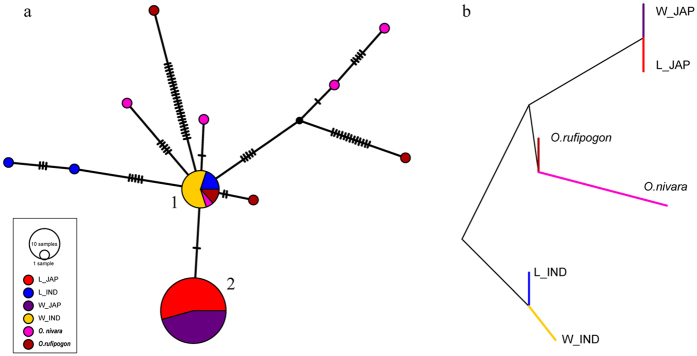
Haplotype network and population splits of the mitochondrial genome suggested by genome wide variations. (**a**) Circle size is proportional to the number of samples within a given haplotype, and dashes between haplotypes represent unobserved, inferred haplotypes. Lines between haplotypes represent mutational steps between alleles. Colors denote rice designation: dark red, *O. rufipogon*; orange and blue, *indica*, red and purple, *japonica*, pink, *O. nivara*. (**b**) Population splits based on the six groups. L/W_IND: *indica* type in landrace or weedy rice, L/W_JAP: *japonica* type in landrace or weedy rice.

Given the observed complex genetic relationship of landrace and weedy rice, and to support our results, we conducted a haplotype network analysis and population split test in the current population. The samples were divided into six groups: *O. rufipogon, O. nivara, landrace_japonica, landrace_indica, weedy_japonica*, and *weedy_indica*. The haplotype network revealed 11 haplotypes in the whole collection, dominated by two common haplotypes that were detected in the majority of landrace and weedy rice (Fig. 5a). Excluding the three wild types with haplotypes similar to the *indica* type, other wild rice accessions, including two *indica* suspected accessions, shared the other minor haplotypes. Generally, haplotype 1 was represented primarily by the *indica* type, whereas haplotype 2 was detected primarily in the *japonica* type. Analysis of population splits among the six groups showed that the *indica* and *japonica* types were separated by wild rice, which also indicating the different origin of *indica* and *japonica* (Fig. 5b).

## Discussion and Conclusions

In this report, we mined mt variation in Korean landrace and weedy rice accessions together with 10 wild rice varieties (*O. nivara* and *O. rufipogon*). Generally, different mt genome variations were detected among different groups and the wild rice is more polymorphic than *indica* and *japonica* (Table 1). Landrace rice has more variations than weedy rice, indicates that landrace rice is more diverse than weedy rice. We also carried out an intersection of different groups, which revealed the wild, *indica*, and *japonica* types shared a huge number of variations (1,068, 22.6% of the total; Supplementary Fig. 1). Similar distributions could be observed in two other groups, with 1,543 variations (32.7% of the total) shared among wild, landrace, and weedy rice (Supplementary Fig. 1); and 1,317 variations (27.9% of the total) shared in *indica* or *japonica* varieties of landrace or weedy rice (Supplementary Fig. 1). Especially among *indica* and *japonica* types, specific variations were more common in landrace than in weedy rice. These results also support that the landrace rice is more diverse than weedy rice.

Evaluation of diversity and genetic distance of landrace and weedy rice revealed that weedy rice has slightly less nucleotide diversity than landrace rice, and that the landrace *indica* and *japonica* types have greater nucleotide diversity than weedy rice (Fig. 2). It is same with our previous result by using nuclear genome of Korea landrace and weedy rice^35^. We found greater genetic distance from wild rice in Korean weedy rice than in landrace rice, with less between landrace and weedy types. The distance between *landrace_indica* and *weedy_indica* types was much greater than that between *landrace_japonica* and *weedy_japonica* types, which suggested that *japonica* is less diverse than *indica*. In addition, genetic distance evaluation suggested that weedy rice was far from wild rice but close to landrace rice, indicated the low breeding level between weedy rice and wild rice.

Population structure analysis suggested that landrace and weedy rice in Korea have mixed genetic backgrounds and cannot be separated in mt genome level (Fig. 3). Not only weedy but landrace rice has admixed population composition, as some varieties covered two or three subpopulation components (Fig. 3b). However, precious little wild rice background was shared in weedy rice accessions, foreboding the distant background of them. PCA of the whole collection indicated that the *indica* and *japonica* types showed admixture in landrace and weedy rice accessions (Fig. 3c,d). It supposed to be that the Korean weedy rice may not from the hybridization between wild and cultivated rice. In common, cultivated rice is also hardly to be hybridized with wild rice (*O. rufipogon* or *O. nivara*) in Korea, since there’s no wild rice in Korean farmers’ field in most cases. Another interesting event we found in the population structure was that most landrace or weedy rice only shared the background within *landrace_japonica* or *landrace*_*indica*, which means the *indica* or *japonica* of weedy rice may from independent cultivated rice (*indica* or *japonica*, respectively). We then conclude that Korean weedy rice not from the wild rice but from the cultivated rice itself.

Phylogenetic analyses using two different methods with different nucleotide substitution models revealed a same phylogenetic structure of all accessions, although the overall tree topology structure was very different (Fig. 4). Three groups (wild rice, *indica*, and *japonica* types) were well illustrated and separated. However, consistent with previous results, landrace and weedy rice could not be clearly seperated both in *indica* and *japonica* subgroups. This supported the complicated genetic background of landrace and weedy rice in mt genome level. As we know, the genome transfer is easily happened between mt genome and nuclear genome^14^,^36^,^37^. Genetic differentiation of nuclear and mt genomes in *indica, japonica* and wild rice exists and may infers the different transfer patterns^36^,^38^. Though we cannot totally avoid all interference from the transfer between mt and nuclear genome, we tried to minimize the bias by removing the most easily transferred region in our study. A set of 965 SNPs, which located at mt highly possible non-transfer region (these regions were identified from the Rice Genome Annotation Project, http://rice.plantbiology.msu.edu/annotation_pseudo_organellar.shtml) were isolated and applied for further evaluation. In addition, the same reference information for the SNPs mining and non-transfer regions characterization were employed, which would also reduce the bias. Population structure and phylogenetic analysis revealed that the results were similar by using all dataset and non-transfer dataset (Figs 3 and 4, Supplementary Fig. 2). Three subpopulations, *indica, japonica* and wild rice can be clustered into the same pattern between the two datasets (*K* = 5 with non-transfer SNPs and *K* = 6 with whole SNPs). The similar result by using whole SNP set and non-transfer SNPs indicated that the transfer pattern has limited impact in current study. This also suggested that the variations based method for phylogenetic or evolution studies is feasible, and using all variations in mt genome is also suit for such analysis with high decent accuracy.

Furthermore, we generated a haplotype network of the whole collection, which was dominated by two common haplotypes, including primarily the *indica* type (haplotype 1) and the *japonica* type (haplotype 2), respectively. Haplotype 1 harbored 15 accessions, and haplotype 2 covered 46 *japonica* accessions (Fig. 5). Haplotypes of landrace and weedy rice were contained within the two major haplotypes, and no additional minor haplotype was found in landrace or weedy rice. These results suggest that Korean landrace and weedy rice do not have unique background with each other at the mt genome level, which hold only the haplotypes distributed between *indica* and *japonica*.

Current report suggests that mt genome–based analyses can be applied in genetic diversity studies, as well as in population genetics and phylogenetic analyses. Outcomes from the rice mt genomes reveal and support the independent origin of *O. sativa* L. and also suggest that Korean weedy rice are more likely to be originated from cultivated rice rather than wild rice. Korean landrace and weedy rice have complicated genetic background and different genetic architecture in mt genome. According to a cytoplasmic-genetic male sterility genes study, the weedy rice in different regions most likely originated from local cultivated rice (*indica* or *japonica*) and the hybrid rice probably has been involved in the evolution of some weedy rice accessions^39^. These results are also consistent with the outcomes from our report. A lack of relationship between weedy and wild rice was characterized in areas where *O. rufipogon* is still present^40^, which also supports the conclusion from current report. It indicates that using mitochondrial genome or CMS (cytoplasmic male sterility) genes are a good method for replenishing the evidence from nuclear genome. Apart from nuclear genome–based genomic and evolution studies, we believe this study of the mt genome will increase our understanding of the genomics and evolution of Korea rice.

## Methods

### Samples and whole-genome resequencing

A core set containing 137 rice accessions with diverse types (landrace, weedy, bred) previously generated from worldwide varieties collected from the National Genebank of the Rural Development Administration (RDA-Genebank, Republic of Korea) using the program PowerCore^41^,^42^ was selected for whole genome resequencing^43^, in which 60 landrace and weedy type rice accessions were isolated for current mt genome analysis (Supplementary Table 9). In addition, 10 wild rice (*Oryza rufipogon* and *Oryza nivara*) from the resequencing set developed by Xu, *et al*.^44^ were also combined in the present study (Supplementary Table 9). Raw data of the 10 wild rice accessions were downloaded from the European Nucleotide Archive (http://www.ebi.ac.uk/ena) under accession numbers SRA023116.

For the 60 landrace and weedy rice accessions, young leaves from a single plant were sampled and stored at –80 °C prior to genomic DNA extraction using the DNeasy Plant Mini Kit (Qiagen). Qualified DNA was used for whole-genome resequencing of the collected rice varieties, with an average coverage of approximately 9× on the Illumina HiSeq 2000 Sequencing Systems Platform (Illumina Inc.).

### Data manipulation and variations

Resequencing raw data of all the accessions were trimmed using Sickle v1.2^45^ to remove low-quality reads followed by alignment using BWA v0.6.2^46^ to the Nipponbare mt genome sequence (Genbank: NC_011033). A Sequence Alignment/Map (SAM) file was created during the mapping and converted to a binary SAM (BAM) file with sorting. Removal of duplicates and addition of read group IDs were performed using Picard Tools v1.88 (http://picard.sourceforge.net/). Final realignment and identification of variation were performed using GATK software v3^47^. The raw variant call format file (VCF format) of all accessions are available as Supplementary Dataset 1. Some scripts and commands used in the software were presented in Supplementary Dataset 2. Statistical analyses were performed to summarize the number and distribution of SNPs and indels based on the HapMap (Haplotype Map) file generated from the VCF file.

### Mt genome variations architecture

Statistics evaluation of mt genome nucleotide diversity (*π*), population genetic distance (*F*st), and Tajima’s *D* in the whole collection and different groups were conducted using VCFtools^48^ across a sliding window 1000 bp in length with a 500-bp step size. Assessments of the calculation were also conducted in different groups to compare the groups divergence.

### Population structure analysis

To estimate individual admixture assuming different numbers of clusters, the population structure was investigated using the program *FRAPPE* (Frequentist Estimation of Individual Ancestry Proportion), which allow for estimation of founding allele frequencies and individual admixture using maximum likelihood estimates^49^. We increased the coancestry clusters spanning from 2 to 6 and ran analysis with 20,000 iterations. With an increasing *K* value range from 2 to 6, we could investigate the individual ancestry events in different clusters. A neighbor-join phylogenetic tree of the landrace, weedy and wild rice was constructed using PHYLIP package (Phylogeny Inference Package v3.695, http://evolution.genetics.washington.edu/phylip.html) with 1000 replicates. Principal component analysis (PCA) was conducted using TASSEL 5^50^ based on the variations that will provide evidence and complement the population structure analysis.

### Mitochondria-based phylogenetic, haplotype network and population splits

ML and BI methods were applied to construct phylogenetic trees for the current collection. Briefly, appropriate nucleotide substitution models were assessed using jModeltest 2.1.7^51^,^52^. To perform phylogenetic analyses, indels were excluded to eliminate potential errors, as the software does not process indels well. A ML tree was conducted using PhyML 3.0^53^ complemented by the best nucleotide substitution model GTR+G selected by the hierarchical LRT (Hierarchical Likelihood Ratio Test)^54^, the Akaike Information Criterion (AIC)^55^ and Bayesian Information Criterion (BIC)^56^ with 1000 bootstrap replicates. A Bayesian tree was constructed using MrBayes 3.2.5^57^ implemented with a Bayesian MCMC search, with two parallel runs of 2 million generations and four chains each. Best-fit model GTR+G were selected according to the Bayesian Information Criterion (BIC)^56^ and compared with the tree generated by the ML method. The phylogenetic tree was displayed and modified using Figtree v1.4.2 (http://tree.bio.ed.ac.uk/software/figtree/). The consensus tree of the bootstrap in the ML method was integrated using Phylip software. Tanglegram for two trees was implemented in Dendroscope^58^ using a Neighbor Net-based heuristic, which is one good way to visualize similarities and differences between two phylogenetic trees side by side connected with lines between taxa that correspond to each other.

A statistical model for estimating the historical relationships among populations, using a graph representation that allows both population splits and migration events was conducted using TreeMix v1.12^59^. In this model, by using genome-wide allele frequency data and a Gaussian approximation to genetic drift, the structure of the graph that showing that relationships between sampled populations and their ancestral populations was inferred. Haplotype network was conducted using PopART (Population Analysis with Reticulate Trees, http://popart.otago.ac.nz) according to the groups in an Integer Neighbor-Joining method.

## Additional Information

**How to cite this article**: Tong, W. *et al*. Genetic variation architecture of mitochondrial genome reveals the differentiation in Korean landrace and weedy rice. *Sci. Rep.* **7**, 43327; doi: 10.1038/srep43327 (2017).

**Publisher's note:** Springer Nature remains neutral with regard to jurisdictional claims in published maps and institutional affiliations.

## Supplementary Material

Supplementary Information

Supplementary Table 1

Supplementary Table 2

Supplementary Table 4

Supplementary Table 5

Supplementary Table 6

Supplementary Table 7

Supplementary Table 8

## Figures and Tables

**Table 1:** Summary and subgroup distribution of the total variations (SNPs and Indels) detected in 60 Korean origin landrace and weedy rice along with 10 wild rice using the mitochondrial genome of *Oryza sativa japonica* as reference.

	Type	Total variation			HQ variation^a^	
Summary	SNPs	4,507			203	
	Indels	210			61	
	Total	4,717			264	
**Subgroup distribution**	**Type**	**NO**. **of Accession**	**Total variation**		**HQ variation**^a^	
			**SNPs**	**Indels**	**SNPs**	**Indels**
	Landrace^b^	30	3,514	174	166	57
	Weedy^b^	30	2,675	170	155	55
	IND type^c^	14	2,400	157	166	57
	JAP type^c^	46	2,696	149	195	53
	Wild^d^	10	2,834	168	241	65

^a^HQ variation: High Quality variations number. Here, it refers to the variations without any missing data and heterozygotes.

^b^The landrace and weedy types were investigated using the program PowerCore.

^c^As defined by the program ADMIXTURE using ~1.6 M nuclear genome SNPs. *indica* (IND type), *japonica* (JAP type).

^d^Wild rice were from the Xu *et al*. (Xu *et al*.)^44^.
