# Supplementary material for: Genetic variation architecture of mitochondrial genome reveals the differentiation in Korean landrace and weedy rice
Source: Sci Rep. 2017 Mar 3;7:43327. doi: 10.1038/srep43327 (PMC5335689; doi:10.1038/srep43327)
Supplement: Supplementary Information [file srep43327-s1.pdf]

Genetic variation architecture of mitochondrial genome reveals the differentiation in Korean landrace and weedy rice

Wei Tong [1,2,*], Qiang He [1,*], Yong-Jin Park [1,3]

[1] Department of Plant Resources, College of Industrial Science, Kongju National University, Yesan, 32439, Republic of Korea

[2] State Key Laboratory of Tea Plant Biology and Utilization, Anhui Agricultural University, Hefei, 230036, Peoples'

Republic of China

[3] Center for crop genetic resource and breeding (CCGRB), Kongju National University, Cheonan, 31080, Republic of Korea

* These authors contributed equally to this work

Correspondence should be addressed to Yong-Jin Park, E-mail: yjpark@kongju.ac.kr

Supplementary information

Supplementary Figures

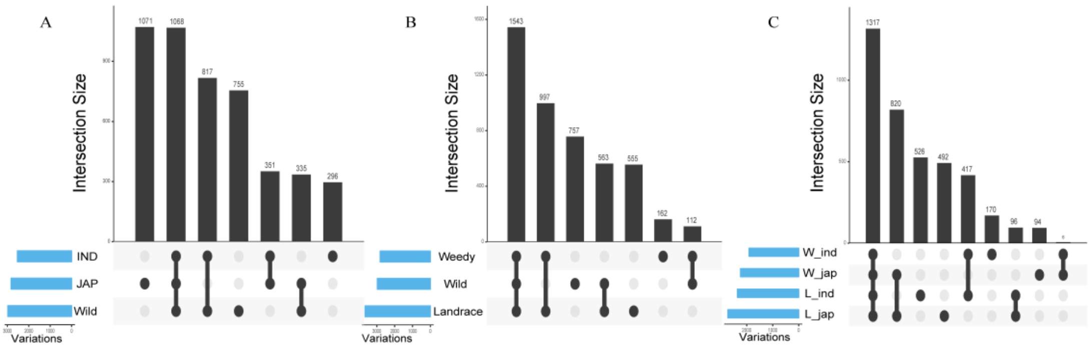

Supplementary Fig 1. Matrix layout for all intersections of different groups. Dark histogram indicated the specific variations in one group or intersected variations among two or more groups. Dark circles in the matrix indicate sets that are part of the intersection. The left set indicated the number of variations in each group. IND: *indica*, JAP: *japonica*, W/L_ind: Weedy and landrace *indica*, W/L_jap: Weedy and landrace *japonica* type.

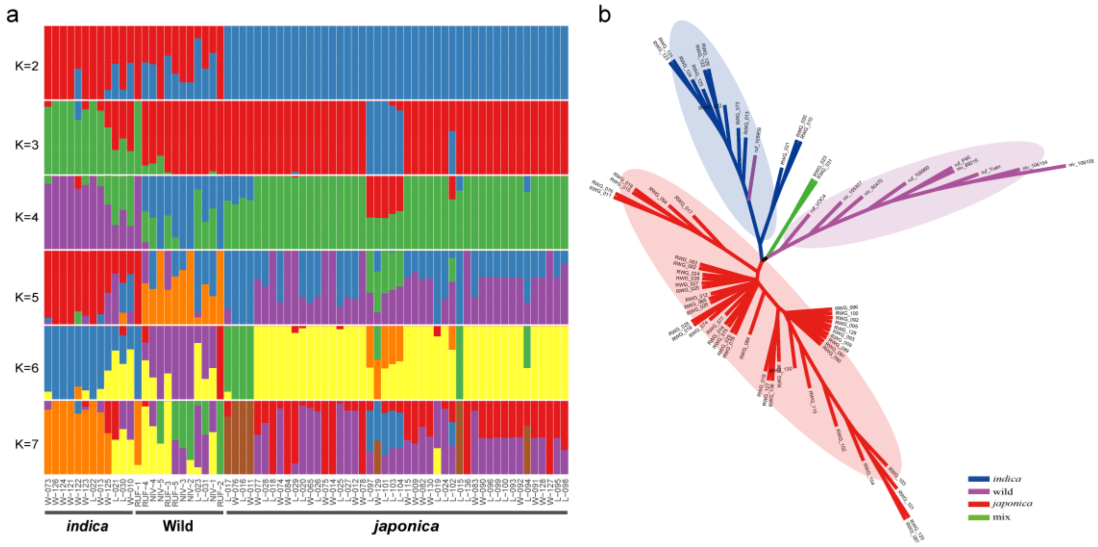

Supplementary Fig 2. Population structure and phylogenetic relationships of the collection using a filtered dataset. This dataset was carried out by removing the SNPs that harbored by the possibly mt insertion in nuclear genome. (a) Population structure clustering of the collection with increasing K value from 2 to 7. (b) A ML tree reveals the phylogenetic relationships of the collection. There main populations were identified. The *indica*, *japonica* and wild rice are marked with different colors.

Supplementary Tables

Supplementary Table 1. Variations identified in 60 Korean landrace and weedy rice accessions. (In single separated file)

Supplementary Table 2. High quality Variations identified in 60 Korean landrace and weedy rice accessions. (In single separated file)

Supplementary Table 3. Regions with few or no variations in rice mitochondrial genome identified in this study.

Supplementary Table 4. Nucleotide diversity of mitochondrial genome in whole collection. (In single separated file)

Supplementary Table 5. Comparison of nucleotide diversity in landrace and weedy rice in mitochondrial genome level. (In single separated file)

Supplementary Table 6. Comparison of nucleotide diversity in landrace&weedy and wild rice in mitochondrial genome level. (In single separated file)

Supplementary Table 7. Comparison of nucleotide diversity in *indica*, *japonica* and wild rice in mitochondrial genome level. (In single separated file)

Supplementary Table 8. Comparison of nucleotide diversity in landrace or weedy *indica* and *japonica* rice in mitochondrial genome level. (In single separated file)

Supplementary Table 9. Information of the 60 Korean landrace and weedy accessions and 10 wild rice accession used in this study.

Supplementary Table 1-2, 4-8 are in separated Excel files.

Supplementary Table 3. Regions with few or no variations in rice mitochondrial genome identified in this study.

NO	Start [a]	End [b]	Length/bp	No. of variations
1	0	43,760	43,760	0
2	94,622	137,917	43,295	0
3	143,763	167,120	23,357	3
4	214,922	222,981	8,059	0
5	273,948	285,414	11,466	1
6	340,731	385,909	45,178	0
7	409,456	416,118	6,662	0
8	421,676	490,520	68,844	2

[a, b] The positions are based on the reference of *O. sativa* L. ssp. *japonica* mitochondrial genome.

Supplementary Table 9. Information of the 60 Korean landrace and weedy accessions and 10 wild rice accession used in this study.

No	Accession name	Origin	Type [a]	Subspecies [b]
Acc-001	Muando	Korea	Landrace	*japonica*
Acc-002	Dadajo	Korea	Landrace	*japonica*
Acc-003	OKCHEONG	Korea	Landrace	*japonica*
Acc-004	Sando	Korea	Landrace	*japonica*
Acc-005	Batnarak	Korea	Landrace	*japonica*
Acc-006	Orido	Korea	Landrace	*japonica*
Acc-007	Saducho	Korea	Landrace	*indica*
Acc-008	SEON	Korea	Landrace	*indica*
Acc-009	Hanyangjo	Korea	Landrace	*admixture*
Acc-010	Inbujido	Korea	Landrace	*japonica*
Acc-011	Beobpanhwa	Korea	Landrace	*japonica*
Acc-012	JANMOCHAL	Korea	Landrace	*japonica*
Acc-013	Pyodo	Korea	Landrace	*japonica*
Acc-014	YULJOJO	Korea	Landrace	*japonica*
Acc-015	Samgyeongjo	Korea	Landrace	*japonica*
Acc-016	BAEKGOGNA	Korea	Landrace	*indica*
Acc-017	MONDONCHALBYEO	Korea	Landrace	*admixture*
Acc-018	Huindadak	Korea	Landrace	*japonica*
Acc-019	Jotajo	Korea	Landrace	*japonica*
Acc-020	Pocheon Jangmang Mebyeo	Korea	Landrace	*japonica*
Acc-021	Dongo Byeo	Korea	Landrace	*japonica*
Acc-022	Seorianjeunbaengi	Korea	Landrace	*japonica*
Acc-023	Neul Byeo	Korea	Landrace	*japonica*
Acc-024	Jwiippari Byeo	Korea	Landrace	*japonica*
Acc-025	Jeongjonghwa	Korea	Landrace	*japonica*
Acc-026	Sodujo	Korea	Landrace	*japonica*
Acc-027	Sando	Korea	Landrace	*japonica*
Acc-028	Bori Byeo	Korea	Landrace	*japonica*
Acc-029	Naengjo	Korea	Landrace	*japonica*
Acc-030	Dudo	Korea	Landrace	*japonica*
Acc-031	Jejubukjeju-2002-99	Korea	Weedy	*japonica*
Acc-032	Jejubukjeju-2002-171	Korea	Weedy	*indica*
Acc-033	Jejubukjeju-2002-340	Korea	Weedy	*japonica*
Acc-034	Jejubukjeju-2002-420	Korea	Weedy	*japonica*
Acc-035	Jejubukjeju-2002-521	Korea	Weedy	*indica*
Acc-036	Incheonkanghwasujip-16	Korea	Weedy	*japonica*
Acc-037	Ssal Byeo 22	Korea	Weedy	*japonica*
Acc-038	Cheongdo-donggok-4	Korea	Weedy	*indica*
Acc-039	Golyeong-2	Korea	Weedy	*japonica*
Acc-040	Golyeong-6	Korea	Weedy	*japonica*

Acc-041	Danyang-7	Korea	Weedy	*japonica*
Acc-042	Danyang-38	Korea	Weedy	*japonica*
Acc-043	Hwaseong-5	Korea	Weedy	*japonica*
Acc-044	Syarebyeo-61-1-B	Korea	Weedy	*japonica*
Acc-045	Syalebyeo-94-1-B	Korea	Weedy	*japonica*
Acc-046	Syalebyeo-163-1-B	Korea	Weedy	*japonica*
Acc-047	Jejubukjeju-2002-115	Korea	Weedy	*japonica*
Acc-048	Jejubukjeju-2002-550	Korea	Weedy	*japonica*
Acc-049	Jejubukjeju-2002-561	Korea	Weedy	*japonica*
Acc-050	Ssalbyeo 16	Korea	Weedy	*japonica*
Acc-051	Chungdo 23	Korea	Weedy	*indica*
Acc-052	Chungdo Hwayang 12	Korea	Weedy	*indica*
Acc-053	Chungdo Hwayang 14	Korea	Weedy	*indica*
Acc-054	Sungju 3	Korea	Weedy	*indica*
Acc-055	Jangsung 1	Korea	Weedy	*indica*
Acc-056	Soonchun 5	Korea	Weedy	*indica*
Acc-057	Daegu Damti 6-2	Korea	Weedy	*japonica*
Acc-058	Guechang 15	Korea	Weedy	*japonica*
Acc-059	Danyang 9	Korea	Weedy	*japonica*
Acc-060	Chungsongaengmi 4	Korea	Weedy	*japonica*
Acc-061	IRGC 105327	India	Wild	*O. nivara*
Acc-062	IRGC 106105	India	Wild	*O. nivara*
Acc-063	IRGC 106154	Vientiane, Laos	Wild	*O. nivara*
Acc-064	IRGC 80470	India	Wild	*O. nivara*
Acc-065	IRGC 89215	Cambodia	Wild	*O. nivara*
Acc-066	IRGC 105958	Indonesia	Wild	*O. rufipogon*
Acc-067	IRGC 105960	Bangladesh	Wild	*O. rufipogon*
Acc-068	VOC4	Nepal	Wild	*O. rufipogon*
Acc-069	P46	China	Wild	*O. rufipogon*
Acc-070	Yuan 3-9	China	Wild	*O. rufipogon*

[a] The type was determined by PowerCore (Kim et al. 2007). [b] Defined by ADMIXTURE (Alexander et al. 2009) when K = 2 using nuclear genome SNP data.
